# Changes in serum-neutralizing antibody potency and breadth post-SARS-CoV-2 mRNA vaccine boost

**DOI:** 10.1016/j.isci.2023.106345

**Published:** 2023-03-06

**Authors:** Manoj S. Nair, Ruy M. Ribeiro, Maple Wang, Anthony D. Bowen, Lihong Liu, Yicheng Guo, Jennifer Y. Chang, Pengfei Wang, Zizhang Sheng, Magdalena E. Sobieszczyk, Alan S. Perelson, Yaoxing Huang, David D. Ho

**Affiliations:** 1 Aaron Diamond AIDS Research Center, Columbia University Vagelos College of Physicians and Surgeons, New York, NY 10032, USA; 2 Theoretical Biology and Biophysics, Theoretical Division, Los Alamos National Laboratory, Los Alamos, NM 87545, USA; 3 Division of Infectious Diseases, Department of Internal Medicine, Columbia University Vagelos College of Physicians and Surgeons, New York, NY 10032, USA

**Keywords:** Biological sciences, Immune response, Immunology, Virology

## Abstract

A better understanding of the durability and breadth of serum-neutralizing antibody responses against multiple severe acute respiratory syndrome coronavirus 2 (SARS-CoV-2) variants elicited by COVID-19 vaccines is crucial in addressing the current pandemic. In this study, we quantified the decay of serum neutralization antibodies (nAbs) after second and third doses of the original COVID-19 mRNA vaccine. Using an authentic virus-neutralization assay, we found that decay half-lives of WA1- and Delta-nAbs were both ∼60 days after second and third vaccine dose. Unexpectedly, the durability of serum antibodies that neutralize three different Omicron subvariants (BA.1.1, BA.5, BA.2.12.1) was substantially better, with half-lives of ≥6 months. A booster dose of the original COVID-19 vaccine was also found to broaden antibody responses against SARS-CoV and four other sarbecoviruses, in addition to multiple SARS-CoV-2 strains. These findings suggest that repeated vaccinations with the COVID-19 vaccine may confer a degree of protection against future spillover of sarbecoviruses from animal reservoirs.

## Highlights

• Single vaccine boost against WA1 spike generated nAb titers to many sarbecoviruses
• Titers decay with similar t_1/2_ against WA1 and Delta variant before and after boost
• Post-boost nAb titers to distinct Omicron variants were lower but decayed slower
• Booster may expand long-lived memory B cells and breadth to conserved spike region

## Graphical abstract

**Figure:**
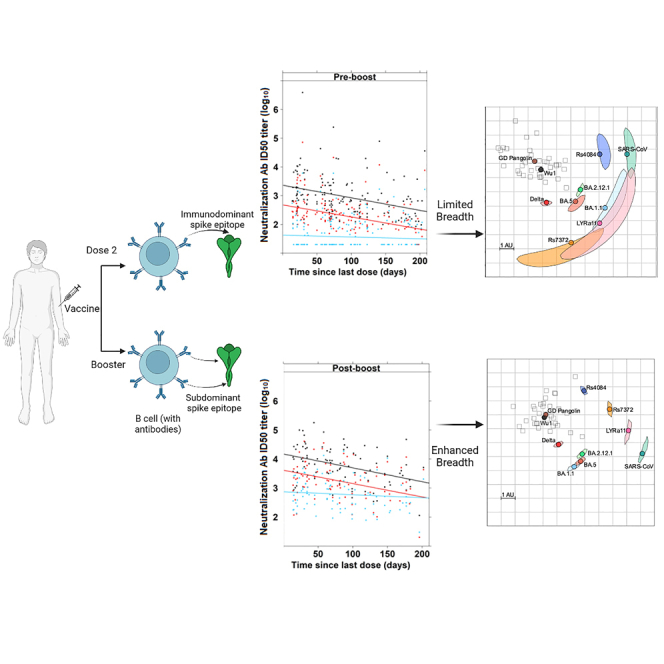

## Introduction

As of December 2022, more than 640 million people have been infected by severe acute respiratory syndrome coronavirus 2 (SARS-CoV-2) with over 6.6 million deaths, according to the World Health Organization (“database:covid19.who.int”). At the same time, about 5.0 billion individuals (>63% of the global population; ourworldindata.org) have been vaccinated with various COVID-19 vaccines, most of which target the parental isolate of the virus that emerged in late 2019. Vaccination efforts, while successful, have slowed primarily due to hesitancy of select populations and the emergence of viral variants of concern (VOCs) that compromised vaccine efficacy.^1^^,^^2^ More recently, multiple lineages of Omicron subvariants have resulted in breakthrough infections^3^^,^^4^ in vaccinated individuals even though vaccines continue to protect against severe disease.^5^^,^^6^ The increased risk of infection observed results from antigenic variation of the virus as well as declining neutralization antibody (nAb) titers in vaccinated individuals. In this study, we quantified the decay of vaccine-induced nAb titers against multiple SARS-CoV-2 variants after the second and third dose of vaccinations (referred to as pre-boost and post-boost, respectively). We also expanded our study to assess the breadth of nAbs against several SARS-CoV-2 variants and related sarbecoviruses that pose as future pandemic threats.

## Results & discussion

**Figure 1:**
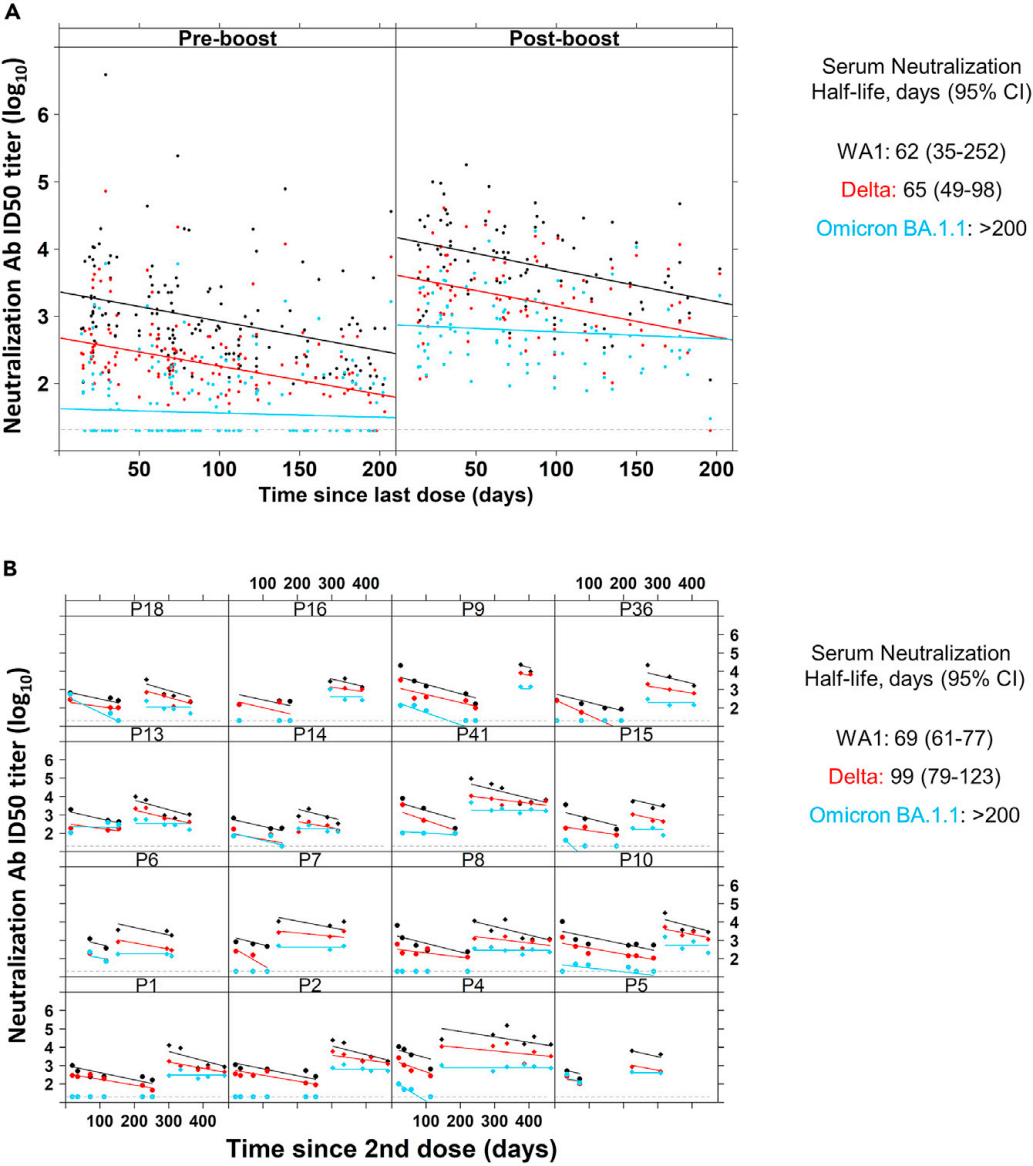
Analyses of the decay of ID50 titers in cross-sectional and longitudinal cohorts (A) Cross-sectional decay of ID50 titers in 149 sera (left panel) collected after second dose and sera from 86 individuals (right panel) collected after first booster dose, up to 200 days post-vaccination. No statistically significant differences between the pre- and post-boost decays for any of the variants. Half-life estimated using best linear model for the decay of the log_10_ ID50 titers. Dashed line in each panel shows the limit of quantitation (LOQ) of the assay. (B) Decay of ID50 titers in 16 individuals followed for up to 200 days following second dose (pre-boost) and their third dose (post-boost) of vaccine. Half-lives of decay of each variant were calculated from the best population estimate (in days) from a mixed-effect model. For the Wuhan and Delta variants there were no statistically significant differences between the pre- and post-boost decays. For the Omicron BA.1.1 variant, most of the titers before the boost were below detection and we could only observe a decay in 6 individuals (t1/2 = 68 days for these), but after the boost there was no decay in any of the individuals, so the half-life is considered greater than the time frame of samples collected (>200 days). Dashed line in each panel shows the LOQ of the assay.

The antigenic variability generated by spike mutations found in VOCs has been extensively studied because of the significant impact that these mutations have on viral escape from polyclonal antibodies.^7^^,^^8^^,^^9^^,^^10^^,^^11^ To underscore the benefits of a booster vaccination, we undertook a systematic examination of the quantitative and qualitative changes in serum antibodies in a cohort of vaccinees who were not infected by SARS-CoV-2 as determined by clinical history and serologic tests for antibodies to the viral nucleocapsid. We first studied the durability of vaccine-induced antibodies in neutralizing the authentic virus of the original Wuhan isolate and two VOCs, Delta and Omicron BA.1.1, by measuring the serum neutralization titers for a period of up to 200 days after the last vaccine dose. We first compared, cross-sectionally, the rate of decline in nAb titers against these variants in 149 serum samples obtained from individuals after their second dose of mRNA vaccination and in 86 serum samples obtained from these individuals after their third dose of mRNA vaccination. We found that, although boosting raised the nAb titers against all variants, it did not significantly alter their decay rates. Pre- and post-boost, the decay of neutralizing titers against WA1 and Delta was similar with mean half-lives (*t*_1/2_) of ∼2 months (Figure 1A). Very few samples possessed a measurable titer to the Omicron BA.1.1 after the second vaccine dose, making it difficult to determine a reliable half-life. The 86 post-boost serum samples had measurable titers to BA.1.1, with an estimated mean decay *t*_1/2_ of >6.6 months, suggesting that the neutralization titers to Omicron, although significantly lower, decayed more slowly than those against parental and Delta strains.

To confirm the robustness of the above findings, we then studied the decay of serum nAbs longitudinally in a cohort of 16 individuals receiving either the Pfizer (N = 14) or the Moderna (N = 2) vaccine and booster doses but were never infected with SARS-CoV-2 based on clinical history and serologic testing (Table S1). Sera collected from these individuals for a period of up to 200 days after the second dose or the booster dose were tested for neutralization against three authentic viruses (WA1, Delta, and Omicron BA.1.1). Consistent with the cross-sectional results, boosting led to higher titers but did not have a significant impact on the decay rates of antibodies that neutralized WA1 and Delta (Figure 1B). After two vaccine shots, the neutralizing titers against Omicron BA.1.1 were generally low again, with 6 of 16 individuals with all measurements below detection and 10 of 16 with very low levels, thereby precluding any meaningful determination of the decay half-life. In contrast, the antibody titers against BA.1.1 post-boost were all above detection but surprisingly very stable over the period of follow-up, with a mean decay half-life ≥6.6 months (Figure 1B).

Since the initial wave of Omicron infections, newer subvariants have gained global prominence, particularly BA.4 and BA.5, which are identical in spike, as well as BA.2.12.1.^11^ To further gauge the protection against these subvariants, we again assessed the serum neutralization against authentic viruses in the aforementioned 16 longitudinal cases after their vaccine boost. The results showed that serum neutralizing titers against BA.5 and BA.2.12.1 decayed nearly identically, with mean half-lives of ∼6 months for both (Figure S1). This observation again suggested that nAbs against Omicron subvariants decay considerably slower than those against WA1 and Delta.

**Figure 2:**
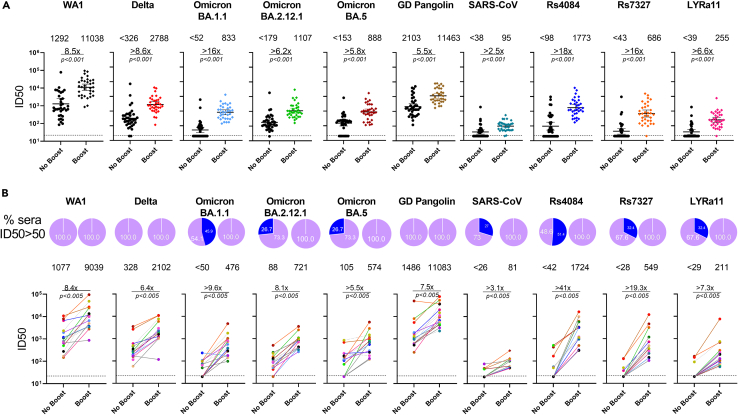
Improved breadth of neutralization after booster vaccine dose (A) Boosted samples dramatically increase the *in vitro* neutralization of the vaccine sera collected between 2 weeks to about 7 months post-booster shot against multiple sarbecoviruses. In addition to increase in potency, improved breadth of neutralization was observed against select sarbecoviruses belonging to clade1a (SARS-1-related coronaviruses) and clade 1b (SARS-2-related coronavirus). Dashed line in each panel shows the LOQ of the assay. Geometric mean ± SEM of each column is shown in figure, and the geometric mean titers of all samples are noted on top of the panel. (B) Matched sample points after second and third dose for 15 individuals demonstrate that the “lack of neutralization titers” seen against many of the clade 1 viruses among these individuals is overcome by the third dose. The pie chart above each panel shows the percentage of sera with ID50 > 50 (in violet) before and after the booster shot. Dashed line in each panel shows the LOQ of the assay. Geometric mean titers of all samples before and after boost are noted on top of the panel.

We next turned our attention to determine the changes in the breadth of the antibody response after vaccine boost. Pre- and post-boost sera from vaccinees (Table S1) were tested for neutralization against the aforementioned five authentic SARS-CoV-2 isolates as well as five pseudoviruses constructed from the spike genes of related sarbecoviruses: GD pangolin, SARS-CoV, Rs4084, Rs7327, and LYRa11 (Figure S2). The results showed that the vaccine boost increased the neutralization potency not only against diverse SARS-CoV-2 isolates by > 5.8- to 16-fold but also against the sarbecoviruses tested by > 2.5- to 16-fold (Figure 2A). The enhanced antibody potency post-boost was seemingly coupled to an expansion of antibody breadth, which could be graphically represented on an antigenic map, showing the compression of the relative antigenic distances between WA1 and other viruses following a third mRNA vaccine dose (Figure S3). To confirm the observed expansion of antibody breadth, we also compared the neutralization of serum samples from a subset of 15 individuals from this cohort at 4–10 weeks before and after boost. Once again, substantial boosting of titers was observed, along with a remarkable widening of breadth against all viruses tested (Figure 2B). In fact, the post-boost serum from all cases demonstrated capability of neutralizing all of the sarbecoviruses tested, again verifying the expansion of antibody neutralization breadth.

It has been previously shown that vaccines can generate B cell memory that initially declines quickly,^12^^,^^13^ but the re-exposure of these B cell clones to the antigen can induce more persistent levels of the antibodies with greater potency and breadth. In this study, we observed a decay in humoral responses to SARS-CoV-2 WA1 and Delta strains with a half-life of approximately 2 months, as has been reported previously.^14^^,^^15^ However, using sera from the same clinical cohort, we noticed a remarkably greater stability in the duration of the antibody neutralizing responses to Omicron BA.1.1, BA.2.12.1, and the BA.4/5 variant, with a half-life of ≥6 months. Given that the half-lives of circulating antibodies are generally very similar, we surmise that our new observation is the consequence of more persistent production of B cells or plasma cells secreting antibody with greater breadth against the Omicron subvariants. Recently, Omicron subvariants BQ.1, BQ.1.1, XBB, and XBB.1 have become dominant in different regions of the world (https://covid.cdc.gov/covid-data-tracker). These subvariants resist serum neutralization by a majority of individuals who have been vaccinated and boosted^16^; therefore, the nAb decay against these subvariants could not be reliably measured.

A recent SARS-CoV-2 vaccine study has shown that increased antigen availability in germinal centers after booster vaccination activates pre-existing memory B cells producing high-affinity antibodies targeting subdominant but conserved spike epitopes by masking the dominant epitopes.^17^ Other studies have observed that a two-dose SARS-CoV-2 vaccination regimen after natural infection increased antibody durability and enhanced breadth across multiple SARS-CoV-2 VOCs.^15^ Cross-sectional analyses have also suggested that a third dose of the original Pfizer or Moderna vaccine increases serum neutralization titers to Beta, Gamma, Delta, and Omicron BA.1 variants.^15^ A booster shot of a COVID-19 mRNA vaccine is known to generate a more diverse repertoire of memory B cells and to yield monoclonal antibodies targeting conserved regions of the receptor-binding domain (RBD) of the viral spike.^18^^,^^19^ In addition, a vaccine boost has been found to proportionally decrease antibodies targeting strain-specific epitopes (RBD Class 1 and 2) and to gradually increase antibodies targeting conserved epitopes (RBD Class 3 and 4).^18^ Our findings on a COVID-19 vaccine boost are consistent with these prior observations, indicating that broadening of serum antibody responses could be elicited without boosting with a new vaccine based on the spike of an emergent variant. What is new, and perhaps unexpected, is the expansion of the nAb breadth extends beyond SARS-CoV-2 variants to include SARS-CoV and other related sarbecoviruses found ubiquitously in animal reservoirs, particularly in bats.^20^ It is possible that the growing herd immunity in the population from repeated COVID-19 vaccinations and infections may be conferring a degree of protection against future spillover of sarbecoviruses from other animals into humans.

### Limitations of the study

Our analysis was restricted to 16 individuals in the longitudinal cohort, and few samples had a shorter follow-up period due to exclusion of volunteers when they turned COVID-positive by nucleocapsid antigen testing. With these limitations, it should be noted that we employed a single exponential model that provided the best statistical support for decay analysis over a power-law or biexponential decay models, especially for minimal decay of titers against Omicron variants.

## STAR★Methods

### Key resources table

**Table:** 

REAGENT or RESOURCE	SOURCE	IDENTIFIER
**Experimental Models: Cell Lines**		

Vero E6	ATCC	Cat# CRL-1586
HEK 293T/17	ATCC	Cat# CRL-11268

**Virus strains**		

VSV-G pseudotype Δ -luciferase	Kerafast	EH1020-PM
WA1	BEI Resources	Cat# NR-52281
B.1.617.2 (Delta)	BEI Resources	Cat# NR-55611
B.1.1.529.1 /BA.1.1 (Omicron)	BEI Resources	Cat# NR-56475
BA.5 (Omicron)	BEI Resources	Cat# NR-58616/NR-58620
BA.2.12.1 (Omicron)	BEI Resources	Cat# NR-56781

**Biological Samples**		

Serum samples from Pfizer BNT162b2 Covid-19 Vaccine trial	Columbia University Irving Medical Center	N/A
Serum samples from Moderna mRNA-1273 Covid-19 ARMOR trial	Columbia University Irving Medical Center	N/A

**Software and Algorithms**		

GraphPad Prism Software v 9.3	GraphPad Prism Software, Inc. www.graphpad.com	N/A
Racmacs1.1.35	https://acorg.github.io/Racmacs/	Smith et al. 2004^21^
Monolix Suite 2021R1	Lixoft (https://lixoft.com/)	Lixoft SAS, 2021
R	https://www.R-project.org/	R Foundation for Statistical Computing, Vienna, Austria.
BioRender	https://biorender.com/	N/A

**Critical Commercial Assays/Reagents**		

FuGENE6	Promega	Cat# E2691
Quikchange II XL site-directed mutagenesis kit	Agilent	Cat# 200522
Luciferase Assay System	Promega	Cat# E1501
Gibson assembly cloning kit	New England Biolabs	Cat# E2611

### Resource availability

#### Lead contact

All requests and queries about this manuscript and its resources should be directed to the Lead Contact, Dr. David D. Ho (dh2994@cumc.columbia.edu)

#### Materials availability

Further information and reasonable requests for resources and reagents should be directed to and will be fulfilled by the Lead Contact, Dr. David D. Ho (dh2994@cumc.columbia.edu).

### Experimental procedures

#### Cell lines

HEK293T/17 (cat# CRL-11268) and Vero E6 cells (cat# CRL-1586) were from ATCC, and they were cultured in 10% Fetal Bovine Serum (FBS, GIBCO cat# 16140071) supplemented Dulbecco’s Modified Eagle Medium (DMEM, ATCC cat# 30-2002) at 37°C, 5% CO_2_.

#### Vaccine sera

The study was approved by the Columbia University Human Research Protection Office Institutional Review Board. All individuals in the study received a BNT162b2 (Pfizer) or mRNA-1273 (Moderna) vaccine and booster doses. Select individuals with BNT162b2 (Pfizer) received a booster dose of Ad26.CoV2.S (J&J) vaccine four to six months after the two primary doses.

#### Authentic SARS-CoV-2 microplate neutralization

The SARS-CoV-2 viruses USA-WA1/2020 (WA1), hCov-19/USA/NY-MSHSPSP-PV29995/2021 (B.1.617.2/Delta variant), hCoV-19/USA/HI-CDC-4359259-001/2021 (B.1.1.529/ Omicron BA.1.1 variant with R346K mutation in spike protein), hCoV-19/USA/COR-22-063113/2022 (BA.5 Omicron variant) and hCoV-19/USA/NY-MSHSPSP-PV56475/2022 (BA.2.12.1 Omicron variant), were obtained from BEI Resources (NIAID, NIH). The viruses were propagated using Vero E6 cells. Virus infectious titer was determined by an end-point dilution and cytopathic effect (CPE) assay on Vero E6 cells as described previously.^10^^,^^22^^,^^23^

An end-point-dilution microplate neutralization assay was performed to measure the neutralization activity of vaccinee sera. Triplicates of each five-fold dilution beginning with a 1:50 dilution of sera were incubated with SARS-CoV-2 at an MOI of 0.1 in EMEM with 7.5% inactivated fetal calf serum (FCS) for 1 hour at 37°C. Post incubation, the virus-antibody mixture was transferred onto a monolayer of Vero E6 cells grown overnight. The cells were incubated with the mixture for ∼70 hrs. CPE was visually scored for each well in a blinded fashion by two independent observers. The results were then converted into percentage neutralization at a given sample dilution, and the averages ± SEM were plotted using a non-linear five-parameter dose-response curve to obtain the ID50 of each sample using GraphPad Prism v.9.3. Statistical analyses between groups was performed using Wilcoxon paired t-test using GraphPad Prism 9.3

#### Pseudovirus production of sarbecoviruses

Spike gene for SARS-CoV and sarbecovirus S genes were codon-optimized for mammalian expression, synthesized by Twist Biosciences, and cloned into the same expression vectors as above by Gibson Assembly (New England Biolabs). Sarbecovirus sequences were retrieved from GenBank under the following accession numbers: GD Pangolin (MT799524), Rs7327 (KY417151), Rs4084 (KY417144), and LYRa11 (KF569996). Recombinant VSV pseudoviruses in which the native glycoprotein was replaced with sarbecovirus S proteins were generated as previously described.^24^^,^^25^ Briefly, human embryonic kidney (HEK) 293T cells (ATCC), at a confluency of 80% were transfected with a S protein expression vector using PEI (1 mg/mL) and cultured overnight at 37°C under 5% CO_2_. Twenty-four hours later, cells were infected with VSV-G–pseudotyped ΔG-luciferase (G∗ΔG-luciferase, Kerafast) at a multiplicity of infection (MOI) of 3 for 2 hours. Afterward, cells were washed three times with 1× PBS, changed to fresh medium, and cultured at 37°C for another 24 hours before supernatants were harvested and clarified by centrifugation at 300*g*for 10 min.

#### Pseudovirus neutralization assay

Pseudoviruses were titrated to standardize the infectivity levels for target cells before setting up neutralization assays. Neutralization assays were then performed as described earlier^24^ by incubating pseudoviruses with five-fold serial dilutions of serum (beginning 1:50 dilution) in triplicate in a 96-well plate for 1 hour at 37°C. For neutralization of SARS-CoV pseudoviruses, Vero-E6 cells were seeded at a density of 4 × 10^4^ cells per well, whereas for neutralization of pseudoviruses derived from other sarbecoviruses, 293T-hACE2 cells were seeded at a density of 1 × 10^5^ cells per well. Luciferase activity was measured using the Luciferase Assay System (Promega), according to the manufacturer’s instructions, 24 hours after cells were added to the pseudovirus and serum. The neutralization curves and IC_50_ values were generated by fitting a nonlinear five-parameter dose-response curve in GraphPad Prism 9.3. Statistical analyses between groups was performed using Wilcoxon paired t-test using GraphPad Prism 9.3.

#### Analyses of ID50 decay

We fitted an exponential decay model to the antibody titers (as values of log_10_ ID50) against the different variants, Wuhan, Delta, Omicron BA.1 and Omicron BA.2. The model was given by

$$W=W_{0}e^{-\lambda_{1}t}$$

$$D=D_{0}e^{-\lambda_{2}t}$$

$$O_{1}=O_{01}e^{-\lambda_{3}t}$$

$$O_{2}=O_{02}e^{-\lambda_{4}t}$$

$$O_{3}=O_{03}e^{-\lambda_{5}t}$$

where W, D, O_1_, O_2_ and O_3_ are the neutralizing antibody titers against the Wuhan, Delta, Omicron BA.1.1, Omicron BA.5 and Omicron BA.2.12.1 SARS-CoV-2 variants, respectively, with *W*_*0*_, *D*_*0*_, *O*_*01*_, *O*_*02*_ and *O*_*03*_, the corresponding baseline value at the time of the first titer measurement. The titers decay at rate λ_1_ for Wuhan, and λ_2_, λ_3_, λ_4_ and λ_5_, for Delta, Omicron BA.1, Omicron BA.5 and Omicron BA.2.12.1, respectively.

The model was implemented in two versions. In the first, for the cross-sectional data (Figure 1A), we fitted all the data for the different variants before and post-boost simultaneously, using the software R,^26^ with the package censReg,^27^ which fits general linear models with censored data. This model included covariates for boost and strain, allowing for different initial values and decay rates for each variant and pre-post-boost. We used the log-likelihood ratio test to compare the different models (for example, the effect of boosting on the decay rates). In the second version, we used the same model with a mixed-effects population approach to fit the longitudinal data. We used Monolix 2021R2 (Lixoft SAS, a Simulations Plus company, Antony, France) to fit the data for all the individuals simultaneously, pre-boost and post-boost, for the Wuhan, Delta and Omicron BA.1 variants (we fitted the Omicron BA.5 and BA.2.12.1 longitudinal data separately, because there was no pre-boost data for these variants). In this model version, λ_2_ and λ_3_ were parameterized as α_1_λ_1_ and α_2_λ_2_, respectively, which allowed us to test whether α_1_ = 1 and/or α_2_ = 1, and thus whether the decay rates were the same for the three different variants with pre- and post-boost. The model also included the effect of boost, both as a random effect and a covariate for the initial titers and the decay rates. We compared the fits using the corrected Bayesian Information Criterion (cBIC), as provided by Monolix. The cBIC is used for model selection, with the lowest cBIC indicating the preferred model. The cBIC takes into consideration the number of parameters used to fit the data and penalizes models with more parameters. We tested other model structures, for example biexponential decay or a power-law decay, but there was no statistical support for these over the simpler single exponential model. For the fits in Figure S1, for the BA.5 and BA.2.12.1 variants, we used a similar approach with parameterization λ_5_ = α_3_λ_4_. But we found that α_3_ = 1 provided the best fit, indicating no difference in the decay rates of antibody titers against these two variants.

#### Antigenic mapping of neutralization data

We utilized antigenic cartography to explore the impact of a third mRNA vaccine dose on antigenic distances between multiple SARS-CoV-1 and SARS-CoV-2 related viruses. Antigenic maps were generated separately using sera samples following two or three doses of an mRNA vaccine. The relative positions of viruses and sera on the maps were determined and optimized as described previously^21^^,^^28^^,^^29^^,^^30^ and such that each antigenic distance unit (AU) corresponds to a two-fold change in ID50. Geometric uncertainty was assessed for all points on both maps using a stress limit of one. Maps were created using the Racmacs package (https://acorg.github.io/Racmacs/)in R. All maps were constructed using 1000 optimizations per map with a dilution step size of 0 and the minimum column basis parameter set to “none.” Serum positions are represented by grey squares, while virus positions are represented by colored circles. Geometric uncertainty was assessed for all points on both maps using a stress limit of one and is illustrated for virus positions as colored regions.

### Quantification and statistical analysis

Neutralization assays were quantified by using a non-linear five parameter regression analysis curve fit to determine the IC50 values that were used in generation of the decay and breadth plots as determined in GraphPad Prism 9.3. Quantification of the decay half-lives were calculated as described in detail in the analyses of ID50 decay section for both the cross-sectional and longitudinal cohorts. Racmacs package was used to define the antigenic cartography maps using the antigenic distance units as described in the antigenic mapping methods section.

## Data Availability

• Data reported in this paper will be shared by the lead contact upon request. • This paper does not report original code. • Any additional information required to reanalyze the data reported in this paper is available from the lead contact upon request.
