# Supplementary material for: Changes in serum-neutralizing antibody potency and breadth post-SARS-CoV-2 mRNA vaccine boost
Source: iScience. 2023 Mar 6;26(4):106345. doi: 10.1016/j.isci.2023.106345 (PMC9987605; doi:10.1016/j.isci.2023.106345)
Supplement: Document S1. Figures S1–S3 and Table S1 [file mmc1.pdf]

iScience, Volume *26*

Supplemental information

Changes in serum-neutralizing antibody potency

and breadth post-SARS-CoV-2 mRNA vaccine boost

Manoj S. Nair, Ruy M. Ribeiro, Maple Wang, Anthony D. Bowen, Lihong Liu, Yicheng Guo, Jennifer Y. Chang, Pengfei Wang, Zizhang Sheng, Magdalena E. Sobieszczyk, Alan S. Perelson, Yaoxing Huang, and David D. Ho

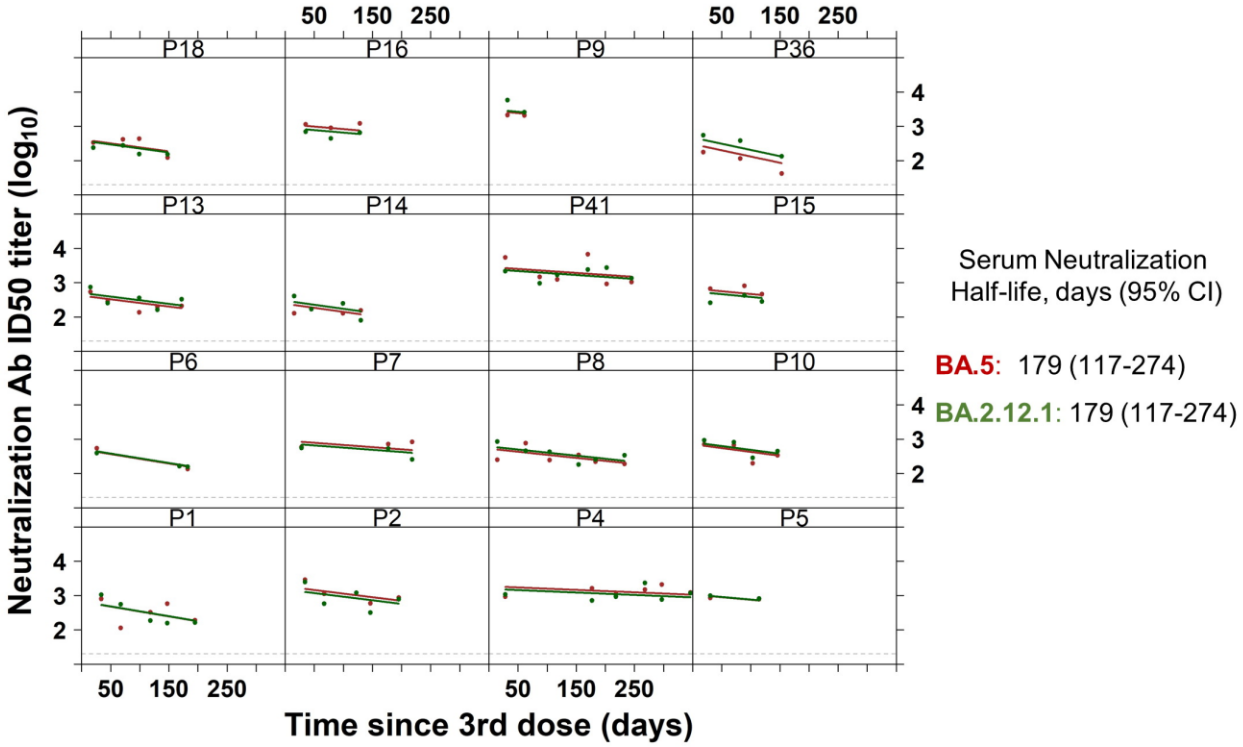

Figure S1. ID50 titers and longitudinal decay of sera against the Omicron BA.2.12.1 variant of SARS-CoV-2 (related to Figure 1)

Longitudinal decay of samples from 16 individuals boosted with parental vaccine shows that the decay rate of ID50 neutralization titers against both BA.5 (red) and BA.2.12.1(green) is around 2-fold slower than against WA1 and Delta variant in the same individuals, but faster than the decay of titers against Omicron BA.1.1. Dashed line in each panel shows the LOQ of the assay.

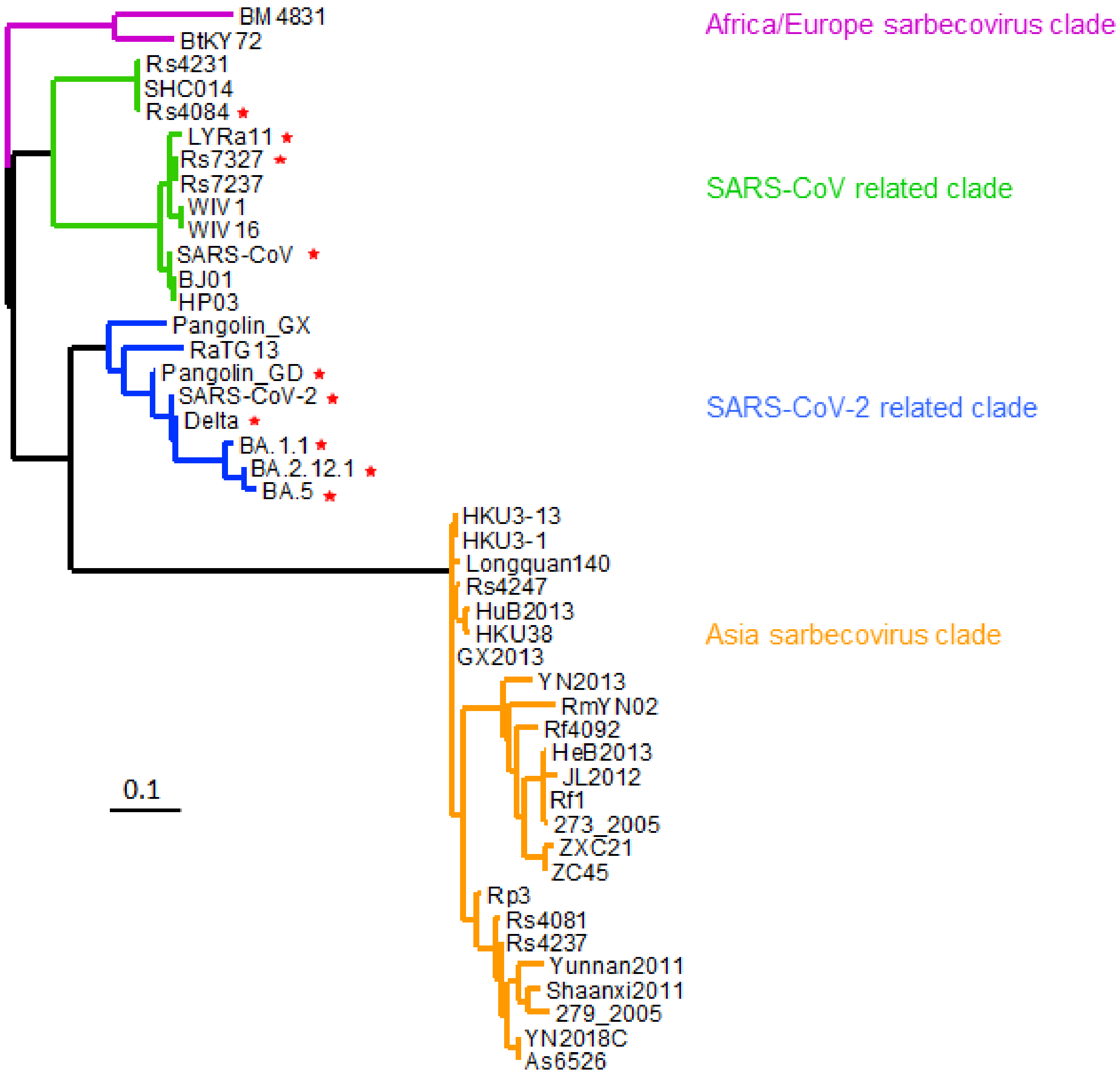

Figure S2. Phylogenetic tree showing distance of RBD sequences among known sarbecoviruses (related to Figure 2)

The collected known sarbecoviruses cluster into four clades. In addition to SARS-CoV, pseudoviruses of three viruses from the SARS-CoV clade and one from the SARS-CoV-2 clade (GX Pangolin) were generated to test breadth of neutralization to vaccine sera (tested viruses are labeled with a red star).

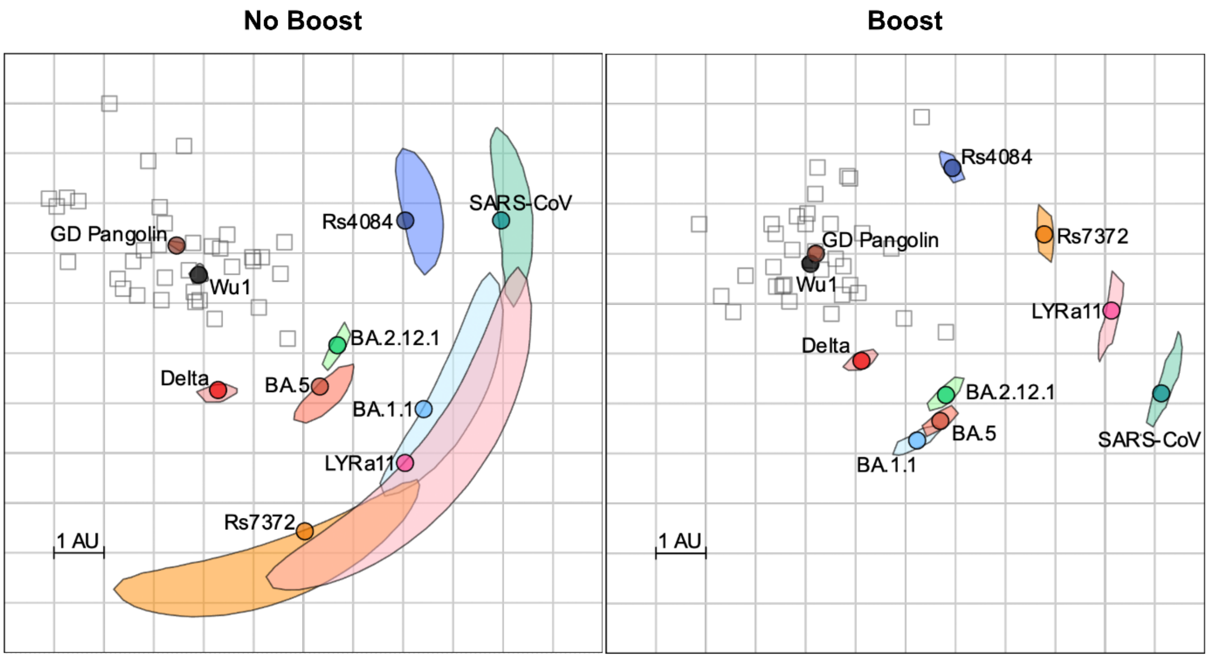

Figure S3. Antigenic cartography showing distance between strains (related to Figure 2)

Antigenic maps generated from the neutralization data show that relative distances between WA1 and GD Pangolin, Rs4084, Rs7327, and Omicron BA.1.1, are compressed following a third mRNA vaccine dose, consistent with a broadening of the humoral response to these viruses. Serum positions are represented by grey squares, while virus positions are represented by colored circles. Geometric uncertainty is illustrated for virus positions as colored regions. Each antigenic distance unit (AU) corresponds to a two-fold change in ID50.

Table S1. Clinical and demographics information of the cohort of individuals studied for longitudinal decay and breadth (related to Figure 1 & 2)

Individuals shown in blue were used in both decay and breadth analyses, those in orange were used in only the decay analysis and the rest in only the breadth analysis. Limiting availability of pre-boost samples prevented their use in longitudinal decay studies against newer variants of Omicron, BA.5 and BA.2.12.1. All samples collected were verified by ELISA to be negative for SARS-CoV-2 NP protein prior to use in all neutralization assays.

ID	Age	Gender	Vaccine type	Booster type	Dose 1	Dose 2	Dose 3
P1	55	Male	BNT162b2	BNT162b2	12/30/2020	1/20/2021	10/15/2021
P10	57	Female	BNT162b2	BNT162b2	1/12/2021	2/3/2021	12/1/2021
P13	48	Female	BNT162b2	BNT162b2	4/8/2021	4/29/2021	11/4/2021
P14	50	Female	BNT162b2	BNT162b2	4/8/2021	4/29/2021	11/4/2021
P15	68	Male	BNT162b2	BNT162b2	1/16/2021	2/6/2021	8/22/2021
P16	58	Female	BNT162b2	BNT162b2	3/14/2021	4/4/2021	12/21/2021
P18	53	Female	BNT162b2	BNT162b2	4/1/2021	4/29/2021	12/1/2021
P19	72	Male	mRNA-1273	mRNA-1273	12/17/2020	2/16/2021	9/8/2021
P2	45	Male	BNT162b2	BNT162b2	12/29/2020	1/19/2021	10/15/2021
P314	38	Female	mRNA-1273	mRNA-1273	12/23/2020	1/20/2021	12/15/2021
P36	61	Male	mRNA-1273	mRNA-1273	12/30/2020	1/20/2021	9/30/2021
P4	63	Male	BNT162b2	Ad26.CoV.2S	12/29/2020	1/19/2021	5/17/2021
P41	66	Female	mRNA-1273	mRNA-1273	1/4/2021	2/1/2021	8/24/2021
P428	46	Female	mRNA-1273	mRNA-1273	12/27/2020	2/2/2021	11/18/2021
P5	30	Male	BNT162b2	Ad26.CoV.2S	12/29/2020	1/19/2021	8/2/2021
P503	41	Female	mRNA-1273	mRNA-1273	12/23/2020	1/20/2021	1/7/2022
P544	40	Female	mRNA-1273	mRNA-1273	12/26/2020	1/23/2021	12/21/2021
P6	69	Male	BNT162b2	Ad26.CoV.2S	12/29/2020	1/19/2021	5/26/2021
P68	70	Female	mRNA-1273	mRNA-1273	12/27/2020	1/23/2021	11/19/2021
P7	29	Male	BNT162b2	Ad26.CoV.2S	12/29/2020	1/19/2021	5/17/2021
P738	57	Female	mRNA-1273	mRNA-1273	12/24/2020	1/21/2021	12/20/2021
P8	64	Female	BNT162b2	BNT162b2	1/3/2021	1/24/2021	9/8/2021
P9	39	Male	BNT162b2	mRNA-1273	1/8/2021	1/29/2021	1/8/2022
